# Biomedical Aerogels in Wound Healing: Therapeutic Strategies and Translational Insights

**DOI:** 10.34133/bmr.0295

**Published:** 2025-12-12

**Authors:** Yukun Liu, Kang Wang, Fangli Gao, Zhikai Xu, Xuan Zhao, Guoyun Wan, Xiangjun Bai, Zhanfei Li, Yuchang Wang

**Affiliations:** ^1^ Department of Plastic and Aesthetic Surgery, Tongji Hospital, Tongji Medical College, Huazhong University of Science and Technology, Wuhan 430030, China.; ^2^ College of Chemistry and Chemical Engineering, Henan Normal University, Xinxiang 4453007, China.; ^3^ Division of Trauma Surgery, Emergency Surgery & Surgical Critical Care, Tongji Hospital, Tongji Medical College, Huazhong University of Science and Technology, Wuhan 430030, China.; ^4^ Sino-German Research Institute of Disaster Medicine, Huazhong University of Science and Technology, Wuhan 430030, China.; ^5^ Department of Emergency and Critical Care Medicine, Tongji Hospital, Tongji Medical College, Huazhong University of Science and Technology, Wuhan 430030, China.; ^6^ Trauma Center, Tongji Hospital, Tongji Medical College, Huazhong University of Science and Technology, Wuhan 430030, China.; ^7^ School of Life Science and Technology, Xinxiang Medical University, Xinxiang 4453007, China.

## Abstract

Wound healing is a complex, highly orchestrated process involving hemostasis, inflammation, proliferation, and remodeling. While acute wounds typically progress through these phases efficiently, chronic wounds—such as diabetic foot ulcers, pressure ulcers, and venous leg ulcers—often stagnate due to persistent bacterial colonization, excessive inflammation, impaired angiogenesis, and reduced extracellular matrix deposition. These pathological features lead to prolonged healing, high recurrence rates, and substantial socioeconomic burdens, which are further exacerbated by global aging, rising diabetes prevalence, and lifestyle-related comorbidities. Conventional wound dressings, including gauze, films, hydrocolloids, and hydrogels, exhibit limitations in infection control, sustained moisture balance, controlled therapeutic release, and adaptability to irregular wound surfaces. Aerogels, a class of ultralightweight, highly porous materials with porosity exceeding 90% and an exceptional surface area, have emerged as promising candidates for advanced wound care. Their unique structure enables superior exudate management, tunable mechanical compliance, and efficient loading of bioactive agents. Composed of inorganic, biopolymeric, or composite matrices, aerogels can be functionalized with antimicrobial nanoparticles, growth factors, or photothermal agents to integrate rapid hemostasis, infection control, immune modulation, and regenerative stimulation within a single platform. However, translational challenges remain, including variability in biodegradation, long-term biocompatibility concerns for certain inorganic systems, high production costs, and scale-up difficulties. This review summarizes recent advances in aerogel-based wound dressings, functionalization strategies, and preclinical evidence while critically analyzing barriers to clinical translation. By bridging multidisciplinary insights, we aim to guide the development of multifunctional aerogel dressings toward precision, intelligent wound care solutions.

## Introduction

Wound healing is a complex and highly orchestrated biological process involving the sequential phases of hemostasis, inflammation, proliferation, and remodeling [1,2]. While acute wounds generally progress through these stages in a predictable manner, chronic wounds—such as diabetic foot ulcers, pressure ulcers, and venous leg ulcers—often fail to transition effectively to subsequent healing stages [3–5]. Such pathological wounds are typically characterized by persistent bacterial colonization, excessive inflammatory responses, impaired angiogenesis, and reduced extracellular matrix (ECM) deposition. These pathophysiological abnormalities ultimately lead to markedly prolonged healing times, high recurrence rates, and remarkable socioeconomic burdens [6–8]. With global population aging, increasing diabetes prevalence, and the rising incidence of lifestyle-related comorbidities, the burden of chronic wounds continues to escalate worldwide [9–11]. In clinical practice, managing these wounds remains a formidable challenge, requiring advanced biomaterials capable of addressing multiple concurrent pathophysiological barriers.

Conventional wound dressings—including gauze, films, hydrocolloids, and some modern hydrogel systems—exhibit substantial limitations in the context of chronic wound management [12]. Many lack sufficient antimicrobial activity, fail to maintain an optimal moist wound environment over extended periods, or offer limited control over drug release kinetics, with release durations often too short to be therapeutically effective [13]. Furthermore, their mechanical compliance is frequently inadequate for conforming to the irregular and dynamically changing wound surfaces. A considerable proportion of existing materials are also unable to effectively modulate the wound microenvironment, particularly in suppressing chronic inflammation and promoting neovascularization—both critical for successful tissue regeneration [14–16]. These shortcomings underscore the urgent need for next-generation multifunctional wound dressings that integrate hemostasis, infection control, immune modulation, and regenerative stimulation within a single platform.

Aerogels are a class of porous materials distinguished by their ultralight weight and exceptionally high porosity and have attracted growing interest in advanced wound care applications [17–19]. Their unique structural features—including porosity exceeding 90%, extremely low density, and exceptionally high specific surface area—enable superior fluid absorption and exudate management while providing an efficient platform for loading diverse bioactive agents [16,20]. Moreover, their tunable pore architecture and mechanical properties allow customized designs tailored to specific wound types and healing stages. The interconnected 3-dimensional (3D) pore network further facilitates gas exchange and moisture balance [21–23]. Aerogels can be derived from a wide range of sources, including inorganic materials (e.g., silica), biopolymers (e.g., cellulose, chitosan, and alginate), and composite systems. They can also be functionalized through various strategies, such as incorporating antimicrobial nanoparticles, immobilizing growth factors, or loading photothermal agents for infection control [24,25]. Despite these advantages, the translational application of aerogels in wound care faces several challenges, including variability in biodegradability, concerns over the long-term biocompatibility of certain inorganic formulations, high production costs associated with supercritical drying, and difficulties in large-scale manufacturing [26,27].

Although previous reviews have discussed the biomedical potential of aerogels [17,20,28], a systematic analysis of their progress, challenges, and opportunities specifically in wound healing is still lacking. This review, therefore, aims to provide a comprehensive summary of aerogel types and functional design strategies for wound repair, critically evaluate their progress in preclinical and animal studies, analyze the key barriers to clinical translation, and outline future perspectives. By integrating the latest cross-disciplinary advances with an in-depth discussion of existing bottlenecks, we seek to provide scientific guidance and innovative insights for the development and clinical adoption of aerogel-based wound dressings, ultimately advancing toward precision and intelligent wound care.

## Types and Characteristics of Aerogels

**Fig. 1.**
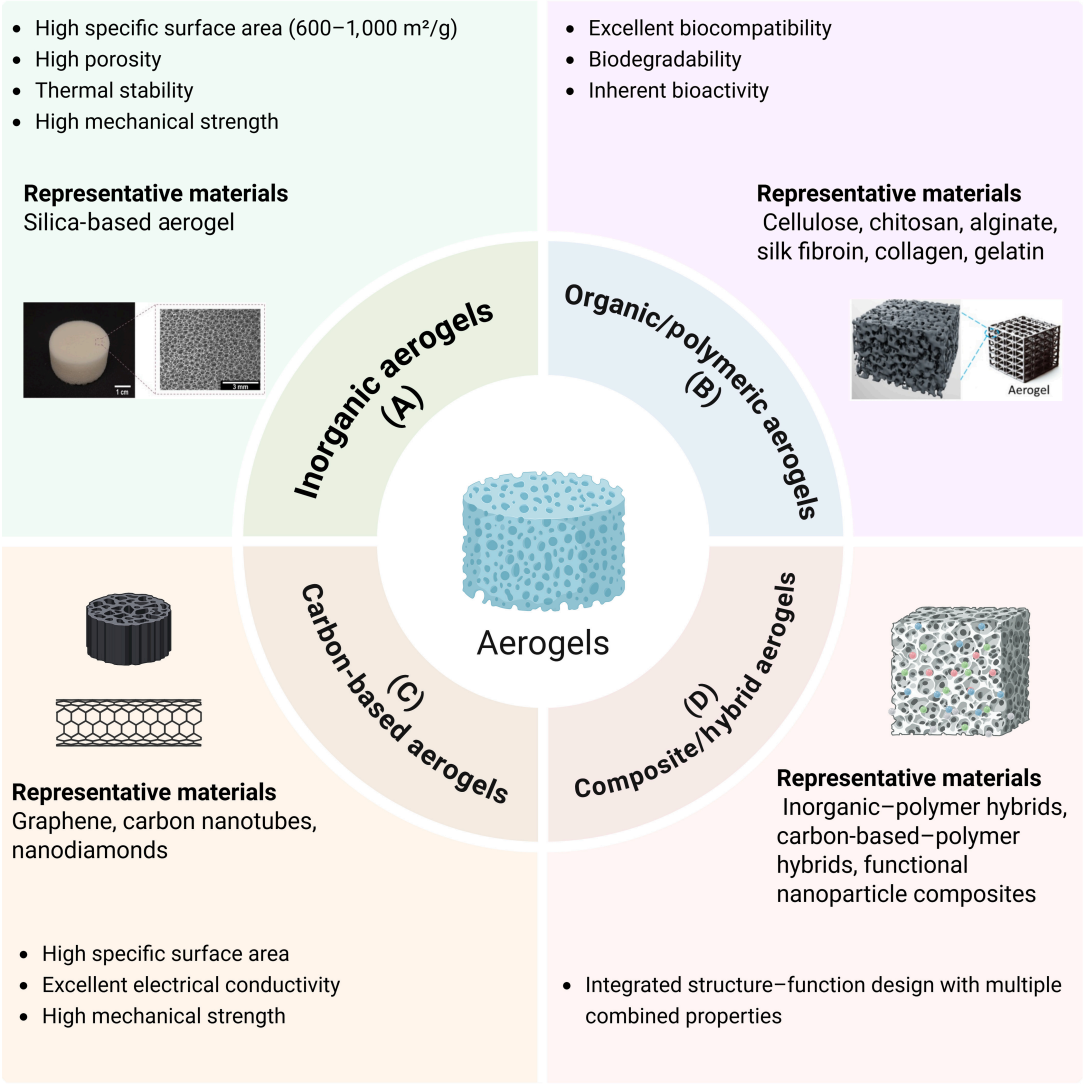
Classification and representative characteristics of aerogels. Aerogels can be broadly categorized into 4 main types: (A) inorganic aerogels, typically silica based, characterized by a high specific surface area (600 to 1,000 m^2^/g), a high porosity, thermal stability, and a high mechanical strength; (B) organic/polymeric aerogels, such as cellulose, chitosan, alginate, silk fibroin, collagen, and gelatin, offering excellent biocompatibility, biodegradability, and inherent bioactivity; (C) carbon-based aerogels, including graphene, carbon nanotubes, and nanodiamonds, noted for their high specific surface area, excellent electrical conductivity, and high mechanical strength; and (D) composite/hybrid aerogels, comprising inorganic–polymer hybrids, carbon-based–polymer hybrids, and functional nanoparticle composites, which integrate multiple structures and functions to achieve combined properties. Representative materials and structural features for each category are illustrated. Created by BioRender.

Aerogels are highly porous materials formed by replacing the liquid phase of a gel with a gas while retaining its 3D network structure [29]. Characterized by an extremely low density, high porosity, and a large specific surface area, aerogels feature an open, interconnected pore architecture that endows them with exceptional physicochemical properties. Owing to these attributes, aerogels have attracted considerable attention for applications in tissue engineering, drug delivery, sensing, and adsorption [17,20]. Depending on their chemical composition, aerogels can be broadly categorized into inorganic types (e.g., silica, titanium dioxide, and aluminum oxide), organic types (e.g., cellulose, pectin, resorcinol–formaldehyde, and polyurethane), carbon aerogels (derived from the pyrolysis of organic precursors), and hybrid or composite aerogels (Fig. 1) [19].

### Inorganic aerogels

Inorganic aerogels, particularly silica (SiO_2_)-based aerogels, are among the earliest developed porous materials and have been widely applied across multiple fields [30]. Their nanoscale pore architecture provides an exceptionally high specific surface area (typically 600 to 1,000 m^2^/g) and a highly controllable pore size distribution, conferring notable advantages in thermal insulation, adsorption, and biomedical applications [31–33]. Silica aerogels combine outstanding thermal stability and mechanical strength with a 3D porous network capable of excellent wound exudate absorption and gas exchange, making them suitable for wound care (Fig. 1A) [31,32].

In wound healing, the ultrahigh porosity and nanoporous structure of silica aerogels enable rapid exudate absorption and maintenance of a moist microenvironment, thereby facilitating cell migration and tissue regeneration [33]. Their large surface area also offers an ideal platform for the loading and controlled release of therapeutic agents such as antimicrobials and growth factors, supporting multifunctional effects including antibacterial action, angiogenesis promotion, and hemostasis [31,32,34,35]. Nonetheless, concerns remain regarding their long-term biocompatibility. Common modification strategies include coating the aerogel surface with biocompatible polymers (e.g., chitosan) to mask potentially harmful moieties and improve mechanical flexibility [31,36].

### Organic/polymeric aerogels

Organic/polymeric aerogels are primarily derived from renewable natural polymers such as cellulose, chitosan, alginate, silk fibroin, collagen, and gelatin [37–39]. These biopolymers not only exhibit excellent biocompatibility and biodegradability but also possess inherent bioactivities that can enhance tissue repair [37,38,40]. For instance, chitosan displays broad-spectrum antimicrobial properties [41], while alginate is widely used in wound management for its outstanding hemostatic capacity [42]. Fabricating these polymers into 3D aerogels allows simultaneous exploitation of the polymers’ intrinsic biological functions and the high porosity and superior moisture absorption of the aerogel architecture (Fig. 1B) [43,44]. The tunable pore structures of such aerogels can provide a biomimetic ECM-like microenvironment, promoting cell adhesion, migration, and proliferation, as well as accelerating epithelialization and collagen deposition [45]. Moreover, incorporation of therapeutic agents within the aerogel scaffold enables multistage wound treatment—combining antibacterial, anti-inflammatory, and proangiogenic effects [46–48]. Despite their excellent biocompatibility, organic aerogels often suffer from poor mechanical stability, high moisture sensitivity, and limited structural durability. Their rapid degradation in moist environments may compromise long-term wound protection [49]. Future optimization should focus on enhancing mechanical robustness and controlling degradation rates through cross-linking or composite reinforcement strategies.

### Carbon-based aerogels

Carbon-based aerogels are porous, lightweight materials composed of carbon nanostructures such as carbon nanotubes, graphene nanosheets, and nanodiamonds, characterized by a high specific surface area, excellent mechanical strength, and superior electrical conductivity (Fig. 1C) [50,51]. These properties confer notable potential for wound healing applications [52,53]. First, their electrical conductivity can be harnessed for electrostimulation to modulate cell proliferation, migration, and differentiation, thus supporting bioelectrical regulation of the healing process [54,55]. Second, carbon-based materials such as graphene and its composites have been shown to promote angiogenesis and tissue regeneration, accelerating wound closure [56]. Additionally, many carbon nanomaterials exhibit inherent antibacterial activity, reducing infection risk and improving treatment safety [57,58]. Importantly, carbon aerogels can serve as carriers for drugs or growth factors, enabling multifunctional synergistic therapy that may include antibacterial effects, proangiogenesis, and photothermally assisted local therapy [59,60]. Although research into carbon aerogels for wound healing is still at an early stage, their multifunctionality and excellent physicochemical properties point to promising future applications. Despite their promising properties, carbon-based aerogels still face challenges for clinical translation. Issues such as potential cytotoxicity, uncertain long-term biodegradability, and difficulties in large-scale production need to be carefully addressed [61].

### Composite and hybrid aerogels

Composite and hybrid aerogels integrate different material systems to achieve synergistic structure–function designs [62]. Inorganic–biopolymer hybrid aerogels, for example, combine the rigidity and structural stability of inorganic components (e.g., silica) with the biodegradability and bioactivity of natural polymers (e.g., collagen and chitosan), addressing the limitations of single-component systems in either mechanical performance or biological functionality (Fig. 1D) [62–64].

Carbon-based hybrid systems (e.g., graphene and carbon nanotubes) endow aerogels with excellent electrical conductivity, enabling integration with electrostimulation therapies to regulate cellular behavior and accelerate tissue regeneration [65–67]. The incorporation of functional nanomaterials—such as silver, copper, zinc oxide, MXene, and photothermal agents—further broadens the therapeutic scope of aerogels, imparting multimodal functionalities including infection control, hemostasis, angiogenesis promotion, and near-infrared (NIR)-triggered localized therapy [68,69]. Although composite and hybrid aerogels offer enhanced functionality, their complexity can lead to challenges in reproducible fabrication, long-term stability, and predictable in vivo behavior. Careful optimization of material ratios, cross-linking, and processing methods will be important to ensure consistent performance and biocompatibility for wound healing applications.

## Functional Design Strategies for Wound Healing Applications

Wound healing is a highly coordinated pathophysiological process comprising 3 overlapping phases—inflammation, proliferation, and remodeling—driven by the interplay of multiple cell types, genes, and cytokines [70,71]. During the inflammatory phase, immune cells migrate to the injury site to clear pathogens and necrotic tissue while releasing cytokines and growth factors that establish a favorable microenvironment for subsequent repair [72,73]. However, fluctuations in pH, inadequate oxygen supply, and bacterial colonization can markedly delay the healing process [74]. Due to their high specific surface area, pronounced porosity, and excellent permeability, aerogel materials can efficiently absorb wound exudates while regulating moisture and pH, thereby providing optimal conditions for tissue regeneration [75,76]. Their porous network structure and modifiable surfaces offer an ideal platform for constructing controlled release systems, enabling the physical adsorption or chemical conjugation of drugs, growth factors, or nucleic acids [37,77]. Multilayer architectures can be designed for stage-specific delivery: outer layers release antibacterial agents rapidly to suppress early-stage infection, while inner layers provide sustained release of pro-healing factors (e.g., vascular endothelial growth factor [VEGF]) to promote angiogenesis and tissue regeneration during later stages [76,78,79]. Furthermore, by integrating stimuli-responsive mechanisms—such as pH, temperature, enzymatic activity, or light—drug release can be triggered specifically within the pathological microenvironment, thereby minimizing systemic side effects and enhancing therapeutic efficacy [80–82].

### Hemostasis

Post-traumatic bleeding is often difficult to control and frequently accompanied by infection risk; thus, the development of highly efficient hemostatic materials is of great clinical relevance for both emergency care and wound repair. In recent years, hemostatic aerogels have demonstrated outstanding advantages in rapid bleeding control, coagulation promotion, and infection prevention, owing to their highly porous architecture and superior liquid absorption capacity [83].

**Fig. 2.**
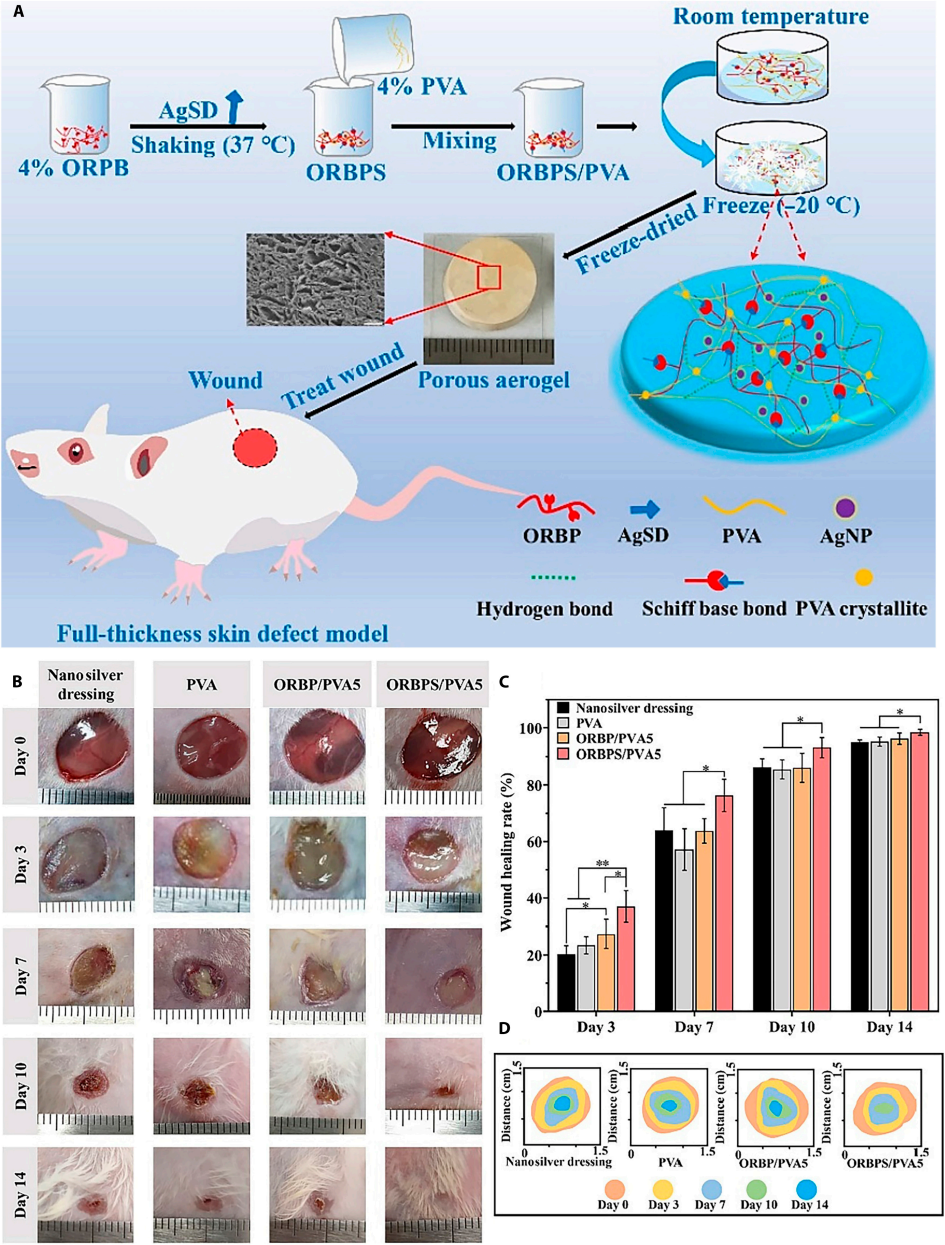
(A) Schematic illustration of oxidized *Bletilla striata* polysaccharide Schiff base/poly(vinyl alcohol) (ORBPS/PVA) composite aerogels’ preparation and application. (B) In vivo promotion of wound healing. Representative photographs of the wound healing process; nanosilver dressing as a control group. (C) Statistics of the wound healing rate. (D) Representative wound closure traces. * indicates *P* < 0.05, and ** indicates *P* < 0.01 [86]. AgSD, silver sulfadiazine; ORBP, oxidized *Bletilla* rhizome polysaccharide; AgNP, silver nanoparticle.

Zheng et al. developed a mesoporous bioactive glass–graphene oxide (GO) composite aerogel featuring a 3D porous structure, high water uptake, and strong hydrophilicity. This material achieved excellent hemostatic performance, shortening the clotting time by 60% and reducing blood loss by 75% compared to medical gauze [84]. Similarly, Borges-Vilches et al. synthesized a superabsorbent aerogel via microwave-assisted methods, capable of absorbing more than 50 times its own weight in water. Incorporation of poly(amidoamine) enhanced surface negative charge and blood adsorption, promoting erythrocyte adhesion and stable fibrin network formation, thereby activating complement and coagulation pathways. The addition of 5% and 10% (w/w) grape skin extract increased total clot formation by 36.6% and 24.5%, respectively [85]. Biocompatibility and bioactivity are also critical for achieving hemostasis while preventing infection. Yan et al. fabricated a freeze-dried, cross-linked hemostatic and antibacterial aerogel by combining oxidized *Bletilla striata* polysaccharide Schiff base with poly(vinyl alcohol) (PVA). The resulting material demonstrated excellent fluid absorption, potent antibacterial activity, and in vivo efficacy in reducing inflammation, promoting angiogenesis and epithelialization, and accelerating wound closure (Fig. 2) [86]. In another example, Tripathi et al. incorporated partially oxidized cellulose nanofibers (T) into a sodium alginate/chitosan (A/C) matrix, further modified with decellularized dermal ECM (E) to produce ACTE aerogels. These materials were noncytotoxic, promoted platelet adhesion and fibrin network formation, and achieved rapid coagulation with stable hemostasis, indicating strong potential for acute hemorrhage control [23]. Collectively, hemostatic aerogels combine structural advantages with multifunctional design to deliver rapid hemostasis, antibacterial and anti-inflammatory effects, and wound healing promotion, making them promising candidates for clinical translation in trauma management.

### Fluid management and exudate control

Chronic wounds, particularly diabetic ulcers, are frequently associated with excessive exudate production and prolonged inflammation [87]. Effective regulation of the wound fluid environment is critical for accelerating healing. To address this clinical challenge, a variety of innovative aerogel materials have been developed to achieve efficient exudate absorption and management.

**Fig. 3.**
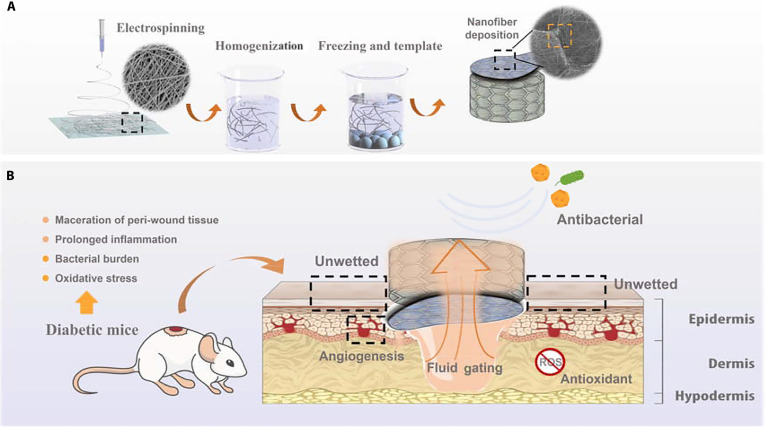
Schematic illustrations of (A) the development process of quaternized chitosan/PVA–polycaprolactone/curcumin (QCS/PVA–PCL/Cur) Janus nanofibrous aerogel and (B) a diabetic wound treated with the Janus nanofibrous aerogel [88]. ROS, reactive oxygen species.

Janus nanofiber aerogels with asymmetric wettability leverage microscale patterning from cell-sized nanofibers to achieve rapid, unidirectional fluid transport without external force, thereby preventing backflow and tissue maceration. Combined antibacterial and antioxidant properties further promote healing; in vivo studies have demonstrated shortened inflammatory phases and accelerated repair of diabetic wounds, along with favorable elasticity and ease of fabrication for potential clinical use (Fig. 3) [88]. Wu et al. advanced this approach by creating antibacterial Janus nanofiber dressings composed of a hydrophilic chitosan aerogel outer layer and a hydrophobic lauroyl chitosan nanofiber membrane inner layer, enabling unidirectional water transfer. These dressings exhibited high liquid absorption (29% to 87%), excellent breathability, and robust mechanical strength (51 MPa), effectively balancing fluid uptake with structural stability [89].

Alginate-based aerogels loaded with Ca–Zn–Ag ions demonstrated outstanding swelling capacity and fluid retention, with calcium ions serving as cross-linkers to promote rapid fluid transport. Zinc ion release levels were comparable to those of conventional dressings, and in vitro studies revealed notable anti-inflammatory activity, highlighting their dual advantages in exudate control and inflammation mitigation [90]. Similarly, electrospun poly(L-lactide-*co*-ε-caprolactone)/acellular dermal matrix composite aerogels with a uniform pore size and favorable mechanical properties provided both hemostatic and exudate absorption capabilities, remarkably enhancing wound repair [44]. Hyaluronic acid/alginate aerogels loaded with zinc oxide nanoparticles exhibited sustained antibacterial activity, effectively preventing infection [85].

From a material design perspective, supramolecular natural deep eutectic solvent/PVA/chitosan aerogels achieved superior water uptake and wettability through optimized micropore architecture, thereby improving exudate environment regulation [91]. More advanced yet is the biomimetic assembled aerogel dressing developed by Guan et al., which utilized a PVA nanofiber framework combined with GO nanosheets to form an ordered porous network. This architecture delivered exceptionally high liquid transport rates and absorption capacities, dramatically enhancing fluid management efficiency for highly exudative wounds [92]. These innovative aerogel designs have successfully achieved high-efficiency exudate absorption and regulation, combined with antibacterial and anti-inflammatory properties, remarkably improving the microenvironment of diabetic and infected wounds and showing strong potential for clinical application.

### Antibacterial and antibiofilm activities

Infection control and biofilm eradication are critical in the management of chronic wounds, particularly in cases involving diabetic foot ulcers, burns, and immunocompromised patients, where biofilm formation substantially diminishes antibiotic efficacy [93–98]. Functionalized aerogels have demonstrated potent broad-spectrum antibacterial activity and effective biofilm disruption through diverse strategies [99–102].

One major approach is the incorporation of metallic and metal oxide nanoparticles (e.g., silver, copper, and zinc oxide) into the aerogel matrix, enabling bactericidal effects via cell membrane disruption or reactive oxygen species generation [103]. For instance, Mao et al. fabricated polylactic acid/gelatin/ZnO nanofiber aerogels via electrospinning and freeze-drying, which exhibited excellent antibacterial performance and biocompatibility. In vivo studies confirmed the accelerated healing of infected wounds, with a higher ZnO content further enhancing therapeutic outcomes [22]. Hu et al. developed zinc oxide/jackfruit aerogels with exceptional water absorption capacity and strong antibacterial activity. Leveraging 3D printing, these aerogels could conform to complex wound geometries, markedly improving healing rates in infected skin wounds [104].

Another strategy employs the cationic biopolymer chitosan, which interacts electrostatically with negatively charged bacterial membranes, increasing permeability and causing leakage of intracellular contents. Batista et al. produced alginate–chitosan composite aerogel fibers with excellent biocompatibility and antibacterial activity, effectively promoting wound closure [21]. Li et al. [105] designed chitosan aerogels loaded with berberine and Ti_3_C_2_T*_x_* MXene, which combined photodynamic and photothermal effects to achieve over 99% bacterial eradication both in vitro and in vivo, thereby accelerating the healing of infected wounds.

**Fig. 4.**
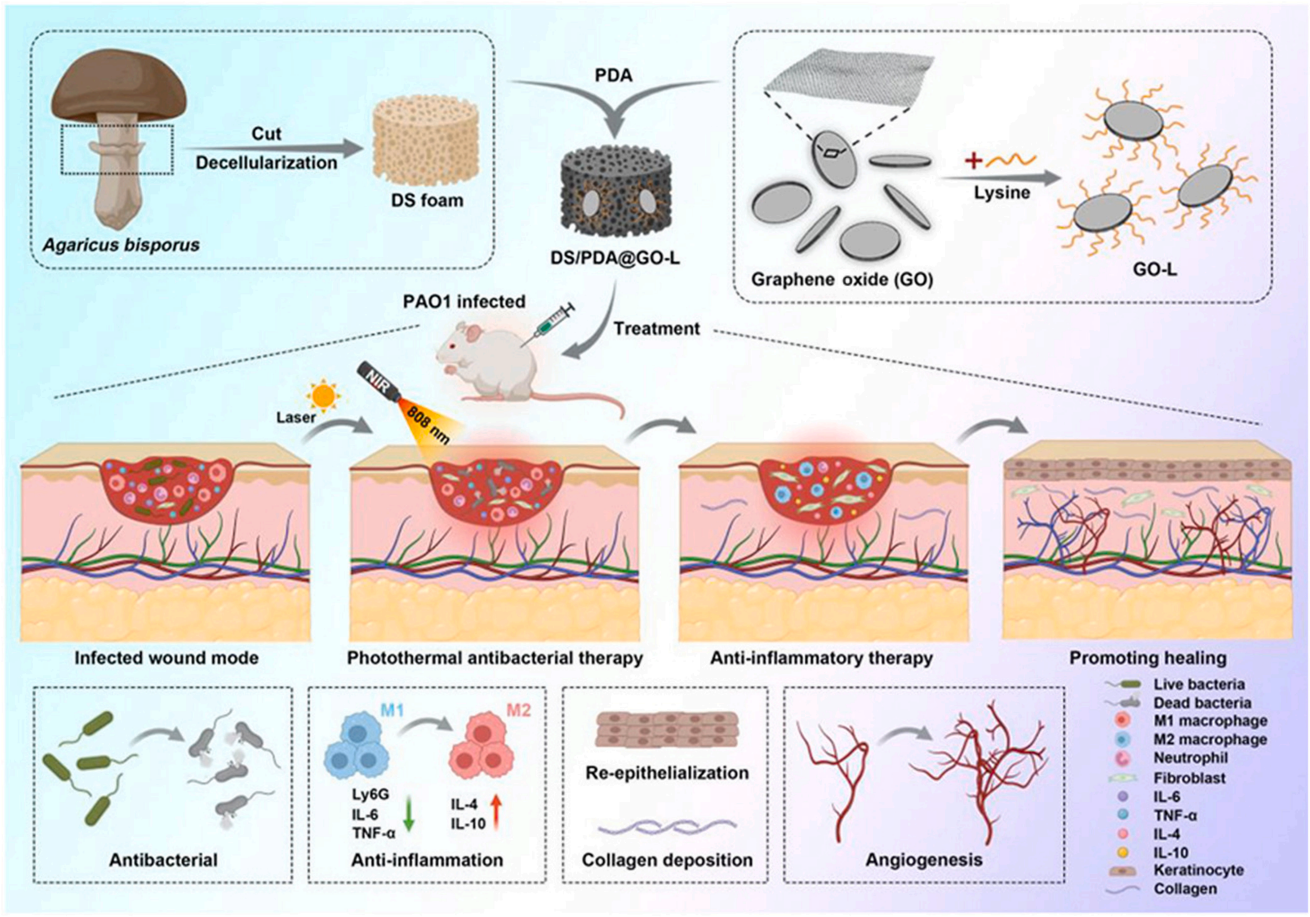
Schematic illustration of the trap–capture–kill strategy for enhanced wound healing. The designed strategy first captures bacteria at the wound site, followed by their neutralization to effectively suppress infection and mitigate inflammation. The reduction of the pro-inflammatory response improves the local wound microenvironment, thereby facilitating extracellular matrix deposition, promoting neovascularization, and accelerating re-epithelialization. These synergistic effects ultimately expedite the wound healing process [106]. DS, decellularized mushroom stem; PDA, polydopamine; GO-L, lysine-modified graphene oxide; PAO1, *Pseudomonas aeruginosa* strain; NIR, near-infrared light; IL-6, interleukin-6; TNF-α, tumor necrosis factor-α; IL-4, interleukin-4; IL-10, interleukin-10.

Photothermal therapy offers a precise and controllable means to eliminate bacteria and disrupt mature biofilms. Zhang et al. [106] engineered DS/PDA@GO-L composite dressings integrating lysine chemotaxis, capillary-driven fluid transport, and NIR photothermal effects, achieving over 95% eradication rates against *Staphylococcus aureus* and *Pseudomonas aeruginosa* (Fig. 4). Lin et al. [107] fabricated chitosan/sodium alginate/calcium peroxide aerogels capable of sustained oxygen release for more than 5 d, demonstrating an ultrahigh antibacterial activity (>99.99%) and strong antioxidative properties. Li et al. reported an alginate aerogel dressing incorporating photothermal, hemostatic, and free-radical-scavenging functionalities via uniform tannic acid–Fe metal–phenolic network formation within an alginate/tannic acid solution, which markedly accelerated healing in methicillin-resistant *S. aureus* (MRSA)-infected murine skin wound models [108]. Similarly, Zhang et al. [109] embedded amino-functionalized MoS_2_ nanosheets into chitosan aerogels, achieving synergistic bactericidal effects through cationic adsorption and NIR-triggered photothermal action.

Emerging smart aerogel dressings integrate wound monitoring with therapeutic functions. For example, anthocyanins from red cabbage were incorporated into carboxymethyl cellulose/PVA aerogels as pH-responsive chromatic indicators, enabling visual monitoring of wound status while maintaining antibacterial activity and biocompatibility [110]. López-Iglesias et al. [111] developed vancomycin-loaded chitosan aerogel beads that exhibited rapid drug release and potent antibacterial effects, ideal for localized infection management. Addressing the complexity of biofilm-associated infections, Yang et al. designed carbonized mushroom aerogels decorated with biometal–organic frameworks (QMOFs-PCMA) that, in combination with photothermal therapy, effectively eradicated biofilms. Additionally, these aerogels scavenged reactive oxygen species, modulated inflammation from a pro-inflammatory to a pro-regenerative state, and promoted granulation tissue formation, re-epithelialization, and angiogenesis [99]. Shen et al. [112] immobilized glucose oxidase and nanozymes within aerogels to establish a cascade catalytic system capable of depleting wound glucose and removing MRSA biofilms, remarkably enhancing antibacterial efficacy.

Multifunctional composite aerogels demonstrate outstanding potential in both infection control and wound repair. Li et al. fabricated sodium alginate oxide/carboxymethyl chitosan/Nb_2_C@Ag/polydopamine (PDA) composite aerogels with remarkable swelling properties, durable antibacterial performance, and excellent biocompatibility. Under laser irradiation, these materials achieved near-complete bacterial inhibition against multiple strains while promoting rapid hemostasis and healing of infected wounds [113]. Furthermore, an oxygen-generating antibacterial xanthan gum–polylactic acid aerogel loaded with dexamethasone enabled sustained oxygen release and controlled drug delivery, synergistically inhibiting bacterial growth and promoting chronic wound healing [114]. In summary, functionalized aerogels achieve effective chronic wound infection and biofilm control through multiple mechanisms, including metal nanoparticle incorporation, natural polymer-based antibacterial action, electro-photothermal synergy, smart responsive monitoring, and immunomodulation. These strategies are driving the development of next-generation multifunctional anti-infective wound dressings with broad clinical translation potential.

### Anti-inflammatory and immunomodulatory effects

Inflammation is a critical regulatory event in the early stages of wound repair; however, excessive or prolonged inflammation can impede healing and exacerbate tissue damage [115,116]. Anti-inflammatory and immunomodulatory aerogels create a favorable microenvironment for tissue regeneration by modulating macrophage polarization, alleviating oxidative stress, and removing excessive wound exudates [3].

**Fig. 5.**
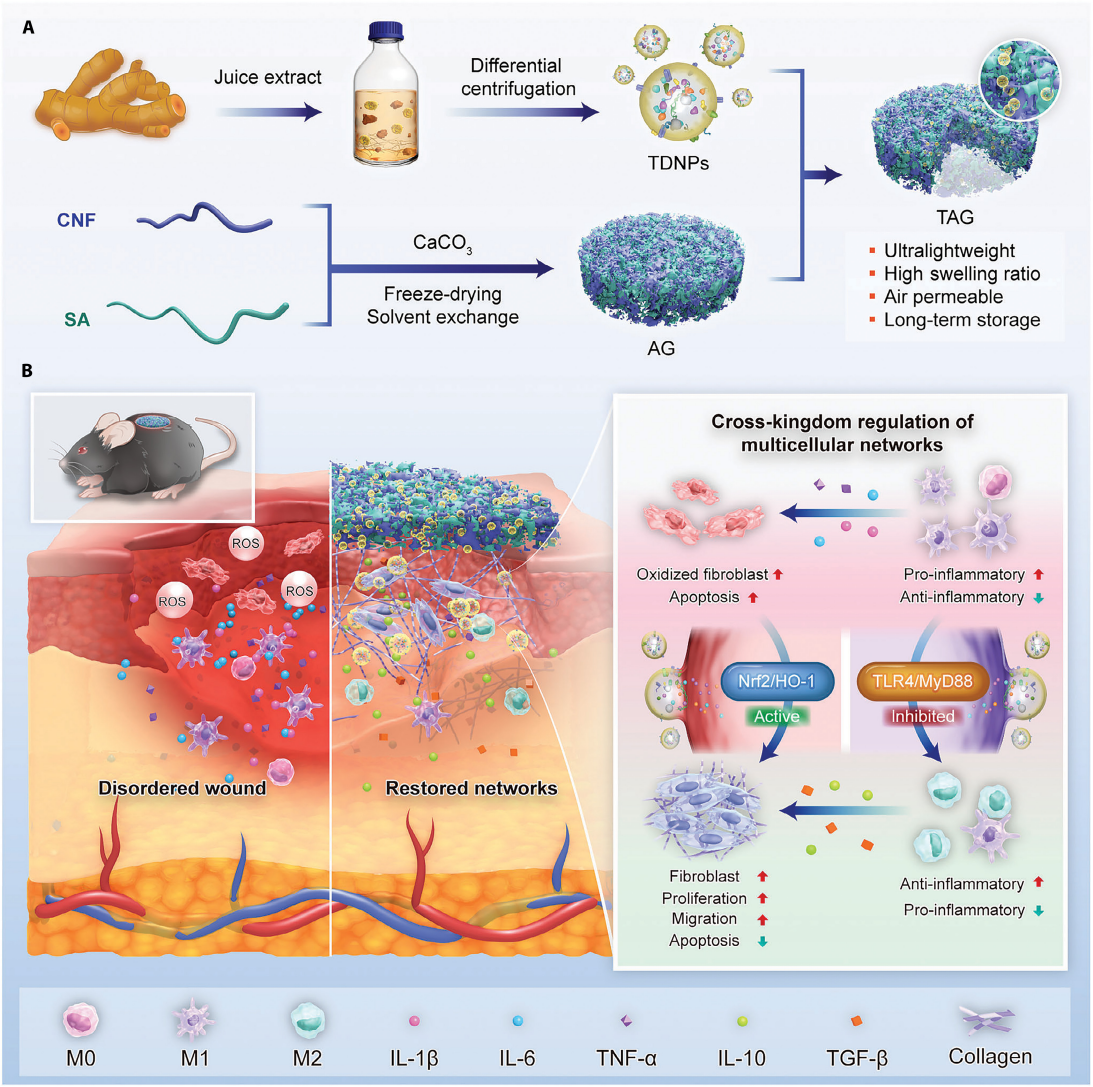
TDNP-loaded aerogel serves as an immunomodulatory dressing by modulating multicellular networks for diabetic wound healing. (A) Isolation of TDNPs and fabrication of AG and TAG dressings. (B) TAG promotes diabetic wound healing by enhancing antioxidant capacity, inhibiting inflammation, and restoring multicellular regulatory networks in the wound microenvironment. TDNPs, turmeric-derived nanoparticles; AG, aerogel; CNF, cellulose nanofiber; SA, sodium alginate; TAG, TDNPs-loaded aerogel (TDNPs@AG); Nrf2, nuclear factor erythroid 2-related factor 2; HO-1, heme oxygenase-1; TLR4, Toll-like receptor 4; IL-1β, interleukin-1β; TGF-β, transforming growth factor-β [117].

One approach leverages natural bioactive compounds, which combine inherent biosafety with multitarget immunoregulatory properties. Wu et al. constructed ultralightweight, highly swellable, and breathable aerogels (AG) based on cellulose nanofibers and sodium alginate, loaded with turmeric-derived nanoparticles (TDNPs) to form TDNPs@AG (TAG). This system alleviated oxidative stress, enhanced fibroblast proliferation and migration, reprogrammed macrophages toward an M2 phenotype, and restored intercellular communication. TAG also exhibited strong tissue adhesion and sustained release capability, markedly enhancing angiogenesis and tissue regeneration in diabetic wounds, thereby highlighting the clinical potential of plant-derived regenerative biomaterials (Fig. 5) [117].

**Fig. 6.**
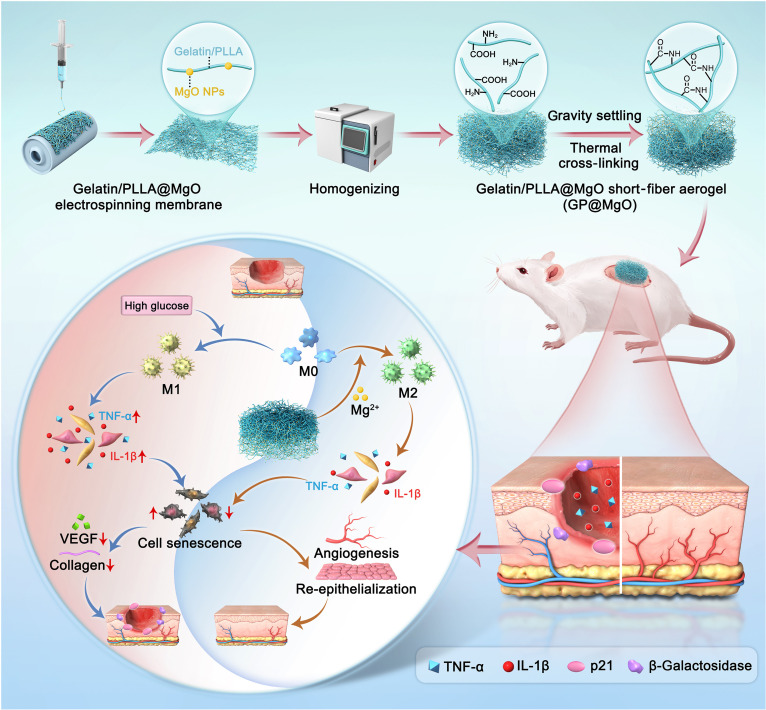
Schematic illustration of asymmetric GP@MgO short-fiber aerogel and therapeutic measures for rescuing immune-induced senescence and accelerating wound closure in diabetic rats [118]. GP, gelatin/poly(l-lactic acid); PLLA, poly(l-lactic acid); NPs, nanoparticles; VEGF, vascular endothelial growth factor.

Alternatively, Liang et al. designed a dextran-based aerogel–hydrogel biphasic system, where the aerogel phase rapidly absorbed and removed wound exudates to create a clean healing environment, while the hydrogel phase—enriched with sodium copper chlorophyllin—provided photodynamic antibacterial effects and incorporated hydrogen sulfide donors to promote M2 macrophage polarization and suppress pro-inflammatory cytokines, collectively improving healing efficiency in infected wounds [46]. Chen et al. fabricated asymmetric short-fiber gelatin/poly(l-lactic acid) (GP) aerogels (GP@MgO) with gravity-induced anisotropic structures for efficient exudate management. Embedded MgO nanoparticles modulated macrophage polarization toward an M2 phenotype and mitigated cellular senescence, notably promoting angiogenesis and re-epithelialization in diabetic wounds (Fig. 6) [118]. Overall, anti-inflammatory and immunomodulatory aerogels—through the controlled release of natural bioactive compounds and interfacial fluid regulation—enable precise intervention within inflammatory environments, offering a promising pathway for systematic treatment of chronic wounds.

### Angiogenesis and tissue regeneration

During chronic wound healing, angiogenesis and tissue regeneration constitute critical stages following the resolution of inflammation [119]. Newly formed blood vessels not only supply oxygen and nutrients to the regenerating tissue but also remove metabolic waste and maintain ECM homeostasis [120]. Aerogel materials, owing to their high surface area, porous architecture, and controlled release capabilities, promote angiogenesis and tissue regeneration via multiple pathways, including the delivery of proangiogenic factors, provision of bioactive ions, construction of stem-cell-friendly scaffolds, and modulation of the immune microenvironment [62,84,121,122].

Certain inorganic components can release proangiogenic ions or small molecules during sustained release. For example, Karan et al. developed high-aspect-ratio copper–cysteine structures (CuHARS) that catalyze nitric oxide (NO) generation, providing both antibacterial activity and stimulation of endothelial-cell-mediated angiogenesis. Incorporation of CuHARS into biomimetic electrospun nanofiber aerogels enabled controlled Cu^2+^ release and continuous NO production, markedly enhancing vascularization in animal models [123]. Similarly, Tan et al. fabricated AuCu@CuO_2_ bimetallic aerogels with quad-enzyme-mimetic activities—namely, peroxidase, superoxide dismutase, catalase, and glutathione peroxidase—and self-oxygenation capabilities. These features alleviated hypoxia, modulated the wound microenvironment, and facilitated angiogenesis and tissue repair in diabetic wounds [124].

The 3D interconnected pore network of aerogels provides favorable adhesion and proliferation sites for reparative cells such as mesenchymal stem cells, improving cell survival and differentiation within the wound. Plant-derived cellulose nanofiber–curcumin aerogels combine high mechanical strength with sustained release, promoting fibroblast migration and angiogenesis without eliciting immune rejection [125]. Chitosan/chondroitin sulfate nanocomposite aerogels, with excellent hydration and biocompatibility, markedly accelerated granulation tissue formation and complete re-epithelialization [126].

Proangiogenic growth factor delivery further enhances the angiogenic potential of aerogels. Chen et al. fabricated VEGF-loaded aerogels (CG-DA-VEGF) by cross-linking chitosan and dopamine-grafted gelatin, achieving stable VEGF release alongside antibacterial, antioxidant, and cytocompatible properties. In vivo, CG-DA-VEGF markedly promoted angiogenesis, re-epithelialization, and collagen deposition [76]. Xue et al. designed multifunctional reduced GO/PDA/Ag/cotton gauze dressings that suppressed inflammation via PDA antioxidant properties and upregulated transforming growth factor-β1, VEGF, and basic fibroblast growth factor expression, achieving 97% wound closure in deep second-degree burn models within 14 d and reducing hemostasis time by 25% [127].

Other strategies involve the synergistic action of natural bioactive molecules with ions or NO. Dharunya et al. [128] developed curcumin-cross-linked collagen aerogels with antiproteolytic activity and proangiogenic functions; their highly organized porous channels improved nutrient diffusion and tissue repair. In summary, aerogel-mediated angiogenesis and tissue regeneration are achieved through growth factor delivery, bioactive ion/NO provision, natural bioactive molecule synergy, and biomimetic ECM scaffolding, enhancing vascular formation, modulating immune responses, and optimizing the local wound microenvironment.

## Clinical Trial Status

Clinical translation of aerogel-based wound dressings remains in its infancy. A review of major clinical trial registries (ClinicalTrials.gov and World Health Organization–International Clinical Trials Registry Platform) and peer-reviewed literature revealed no registered randomized controlled trials of aerogel dressings specifically for wound healing.

The most prominent example is a Bayer-sponsored trial for venous leg ulcers (NCT00998673), investigating silica gel fiber (SGF) dressings versus standard care in terms of healing time. However, this trial was terminated, limiting the interpretability of efficacy outcomes. Despite termination, 2 published studies report clinical use of SGF: (a) a comparative study suggesting favorable performance and safety in venous leg ulcer treatment [129] and (b) a randomized noninferiority trial involving 130 patients showing SGF efficacy comparable to that of alginate dressings, with reduced dressing changes and improved pain relief and patient satisfaction [130]. These data are limited to venous leg ulcers with short- to mid-term follow-up, leaving long-term efficacy unverified. Numerous polymer-based bio-aerogels (alginate, chitosan, cellulose, agarose, and composites) have shown promising preclinical outcomes, including hemostasis, exudate management, antibacterial, and anti-inflammatory effects. As of 2025 August 1, no aerogel formulations have progressed to human trial registration, indicating that the research pipeline remains largely preclinical. Future efforts should focus on multicenter randomized controlled trials encompassing diabetic foot ulcers and infected chronic wounds, standardized endpoints, and improved transparency in device registration and postmarket surveillance.

## Challenges and Future Perspectives

Despite the remarkable potential of aerogels in wound healing, clinical translation faces several technical and regulatory challenges. First, biodegradability directly affects long-term safety and metabolic clearance. Natural polymer-based aerogels (e.g., chitosan, gelatin, and cellulose) generally degrade into nontoxic, easily absorbed or excreted products, although degradation rates vary across pathological conditions [131]. In contrast, the long-term accumulation and chronic toxicity of inorganic aerogels (e.g., silica and alumina) remain poorly understood, particularly in immunocompromised individuals. The degradation mechanisms of composite aerogels are even more complex, requiring integrated in vitro and in vivo assessments to optimize material design. A comprehensive safety evaluation should encompass chronic toxicity, immunogenicity, and hypersensitivity to ensure no adverse effects during long-term use.

Current aerogel fabrication often relies on energy-intensive processes such as supercritical drying or freeze-drying, limiting large-scale production and increasing costs. Maintaining uniform macrostructures and controllable microarchitectures at scale remains challenging. Emerging approaches, including continuous drying, ambient-pressure low-temperature methods, 3D template-assisted fabrication, and automated production lines, are needed to improve yield, reproducibility, and commercial viability.

Advanced manufacturing technologies, such as 3D printing, microfluidics, electrospinning, and layer-by-layer assembly, offer opportunities to integrate multifunctional properties, including antibacterial activity, proangiogenic factor delivery, and stimuli-responsive drug release. Such approaches can replicate ECM-like architectures, enable spatially defined therapeutic loading, and facilitate personalized wound therapies.

Clinically, aerogels intended as medical devices or combination products face complex regulatory pathways that differ across regions. At present, clinical research on aerogels for wound healing remains at an early stage, with no large-scale trials yet completed. Looking forward, research should focus on developing aerogels with integrated multifunctional capabilities—combining antibacterial, anti-inflammatory, proangiogenic, and stimuli-responsive properties—while prioritizing sustainable manufacturing using renewable materials and low-energy processes. Incorporating real-time monitoring of key wound parameters, such as pH, humidity, and temperature, could further enhance therapeutic precision. Successful clinical translation will require close collaboration among researchers, industry partners, and clinicians to effectively transform laboratory innovations into patient-centered wound care solutions.

## Conclusion

Aerogels, characterized by ultrahigh porosity, tunable mechanical properties, and excellent hygroscopic and drug-loading capacities, have emerged as a highly promising platform for advanced wound healing. Preclinical studies have demonstrated that aerogels can maintain a moist wound environment, efficiently manage exudates, control infection, and enable multifaceted interventions in chronic and complex wounds through carefully engineered structural and functional designs. Beyond these technical advantages, aerogels offer unique opportunities to integrate smart functionalities, such as stimuli-responsive drug release, electrostimulation, and real-time wound monitoring, paving the way for precision wound care.

Despite these promising findings, clinical translation remains limited by incomplete understanding of long-term biodegradation, challenges in scalable and reproducible fabrication, and complex regulatory requirements. Addressing these barriers will require interdisciplinary efforts combining materials science, bioengineering, and clinical insights. In particular, systematic evaluation of biocompatibility, in vivo efficacy, and patient-centered outcomes will be critical to ensure safe and effective application. With continued advances in green and scalable manufacturing, coupled with rigorous preclinical and translational research, aerogels are poised to become next-generation, high-performance wound healing materials, holding transformative potential in both routine care and precision medicine contexts.

## Ethical Approval

No individual personal data are included in the study.

## Data Availability

The data that support the findings of this study are available from the corresponding authors upon reasonable request.

## Supplementary Materials

Supplementary 1 Graphical Abstract
