# Supplementary figures and images for: Biomedical Aerogels in Wound Healing: Therapeutic Strategies and Translational Insights
Source: Biomater Res. 2025 Dec 12;29:0295. doi: 10.34133/bmr.0295 (PMC12698940; doi:10.34133/bmr.0295)

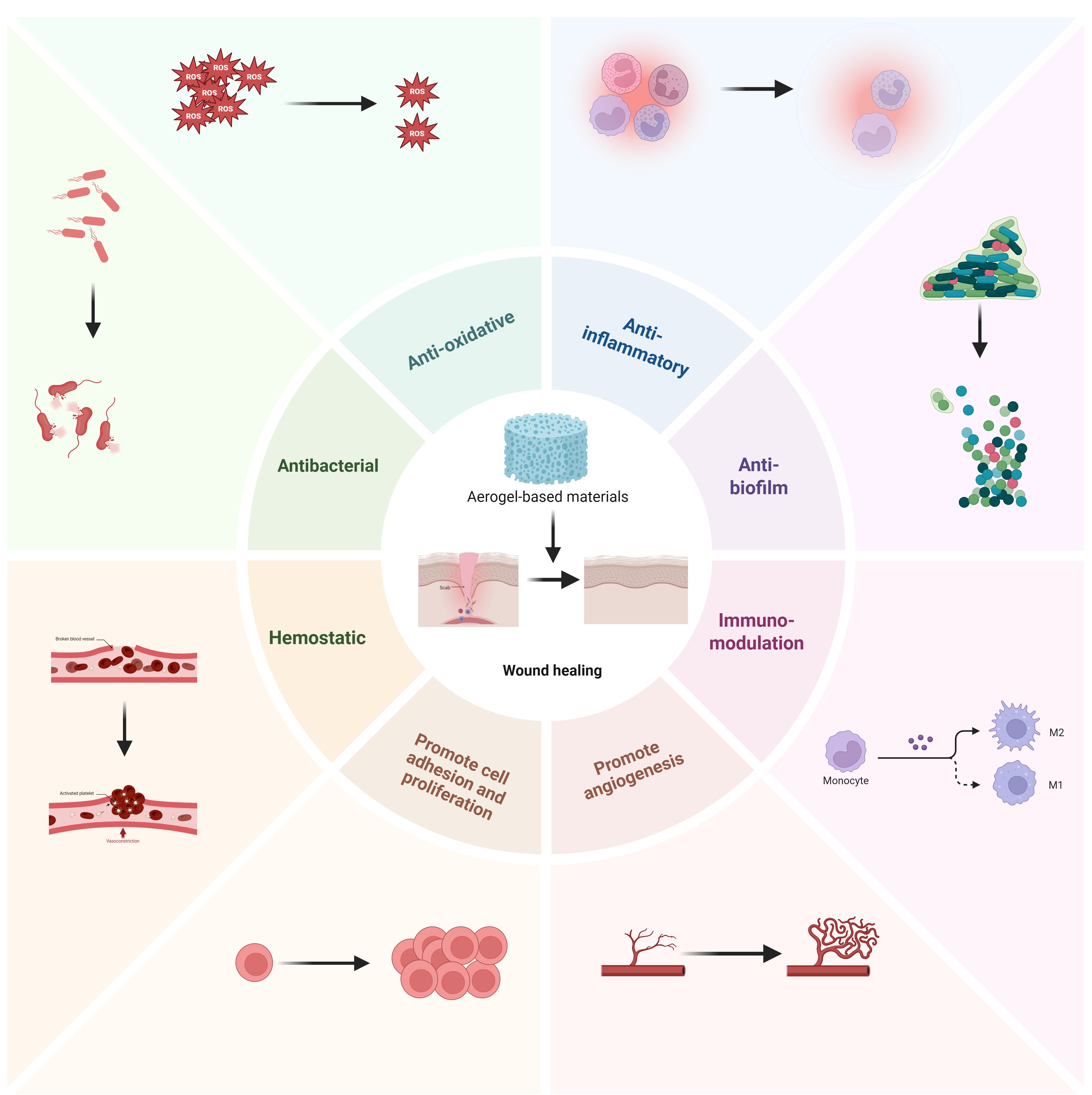

Supplement: Supplementary 1 — Graphical Abstract [file bmr.0295.f1.zip › Graphical abstract.jpeg]
